# X-ray diffraction data and analysis to support phase identification in FeSe and Fe_7_Se_8_ epitaxial thin films

**DOI:** 10.1016/j.dib.2019.104778

**Published:** 2019-11-06

**Authors:** Sumner B. Harris, Renato P. Camata

**Affiliations:** Department of Physics, University of Alabama at Birmingham, Birmingham, AL 35294, USA

**Keywords:** Double epitaxy, Iron selenide, Fe7Se8, FeSe, Iron-based superconductor

## Abstract

X-ray diffraction (XRD) data and analysis for epitaxial iron selenide thin films grown by pulsed laser deposition (PLD) are presented to support the conclusions in the related research article “Double epitaxy of tetragonal and hexagonal phases in the FeSe system” [1]. The films contain β-FeSe and Fe_7_Se_8_ phases in a double epitaxy configuration with the β-FeSe phase (001) oriented on the (001) MgO growth substrate. Fe_7_Se_8_ simultaneously takes on two different epitaxial orientations in certain growth conditions, exhibiting both (101)- and (001)- orientations. Each of these orientations are verified with the presented XRD data. Additionally, XRD data used to determine the PLD target composition as well as mosaic structure of the β-FeSe phase are shown.

**Table:** Specifications Table

Subject area	*Physics, Materials Science*
More specific subject area	*Double epitaxy, Iron-based superconductor, FeSe, Fe*_*7*_*Se*_*8*_*, Iron chalcogenides, Pulsed laser deposition*
Type of data	*XRD data*
How data was acquired	Philips X'Pert-MPD
Data format	*Raw and analyzed*
Parameters for data collection	X-ray tube parameters: 45 kV, 40 mA θ-2θ range and scan rate: 2θ = 25–75°, 0.05°/s Rocking curve range and scan rate: 2θ = 14.5–17.5°, 0.1°/s 2θ range and scan rate: 2θ = 10–80°, 0.1°/s
Description of data collection	XRD is presented to support epitaxial orientation, crystal phase identification, PLD target composition, and mosaic structure in epitaxial thin films.
Data source location	*Birmingham, Alabama USA*
Data accessibility	*Data files have been uploaded alongside article*
Related research article	*S.B. Harris and R.P. Camata, Double epitaxy of tetragonal and hexagonal phases in the FeSe system, J. Cryst. Growth, 514, 2019, 54–59* [1].

**Table:** 

Value of the Data • The data provide insight on how to identify crystal phases in epitaxial thin films that share the same fundamental structure but differ in their the vacancy superstructure or lack thereof. • Similar measurements and analysis procedures as shown in this article can be used to aid in the phase identification of other closely related crystal structures in single crystal samples. • The data provides important supplementary information to the related research article.

## 1 Data

The complex binary phase diagram of the Fe–Se system poses several challenges for researchers in the fields of single crystal and thin film growth of FeSe and related compounds. The crystal phase of greatest interest in recent years is tetragonal β-FeSe (space group *P4*/*nmm*), due to intense interest in its superconducting properties, and has been successfully isolated in thin films across a broad range of conditions [2]. Several hexagonal iron selenide variants lie in close proximity to β-FeSe in the Fe–Se phase diagram. Stoichiometric δ-FeSe forms with the NiAs structure (space group *P6*_*3*_*mc*) at high temperatures and Fe_7_Se_8_ can form concurrently with β-FeSe in the presence of a slight excess of Se at lower temperatures [3,4]. This property, in combination with proper choice of substrate, can be taken advantage of to grow epitaxial thin films which contain two phases of iron selenide in a configuration known as double epitaxy. Double epitaxy may be useful to modulate the properties of the grown materials by introducing many interfaces at fixed angles with respect to each other as well as to the substrate.

Fe_7_Se_8_ has a fundamental NiAs-type lattice, identical to δ-FeSe, but with ordered Fe vacancies which take on several different arrangements depending on the synthesis technique and annealing times and temperatures [5]. The two Fe vacancy orderings most commonly observed and with relevance to the present work are the 3*c* and 4*c* structures of Fe_7_Se_8_. Ordered Fe vacancies in these structures repeat along the *c*-axis at increments that are three (3*c*) or four (4*c*) times the fundamental NiAs-type *c* lattice constant. The 3*c* unit cell is defined with lattice constants *A* = 2*a* and *C* = 3*c* while the 4*c* unit cell is defined by *A* = $\sqrt{3}$ *B*, *B* = 2*a*, and *C* = 4*c* where *a* and *c* are the shared NiAs-type lattice constants [5].

**Fig. 1:**
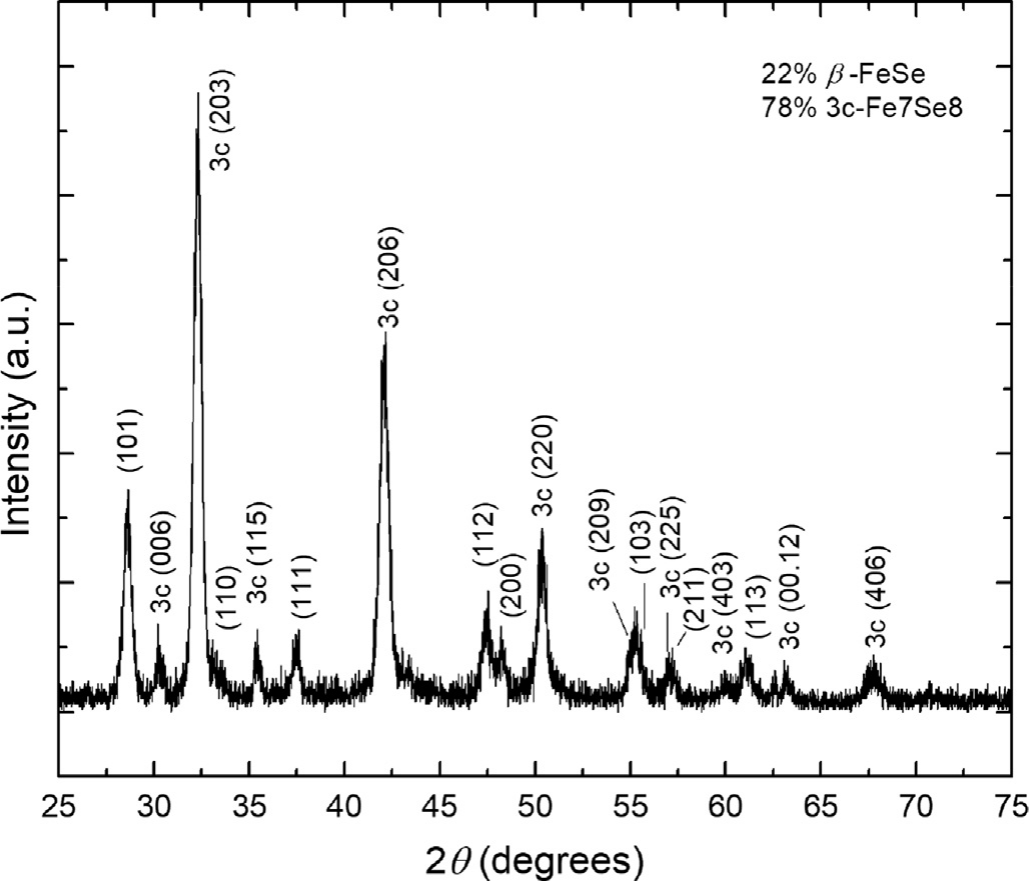
Undoped FeSe target XRD shows a mixture of 22% β-FeSe and 78% 3c-Fe_7_Se_8_.

**Fig. 2:**
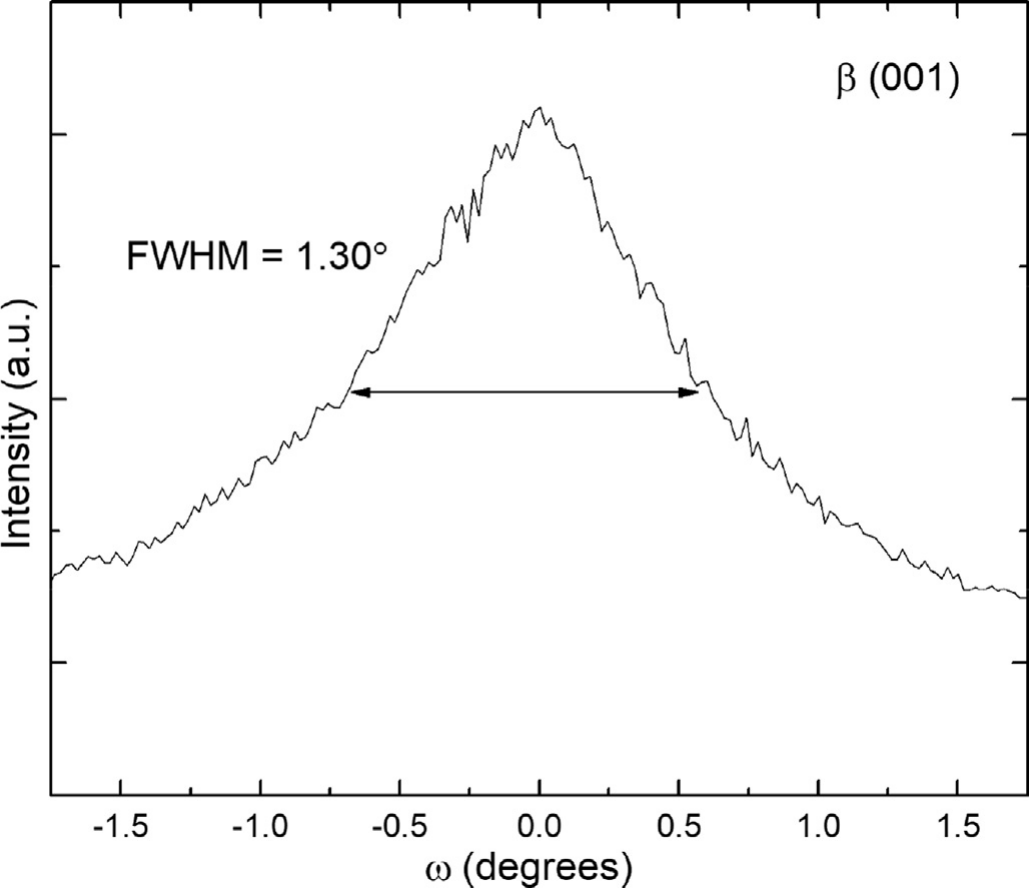
Rocking curve of β-FeSe (001) peak for film grown at 500 °C and 3.4 J/cm^2^.

In the related research article [1], epitaxial thin films were grown by pulsed laser deposition (PLD) using a target formed of a mixture of β-FeSe (22%) and 3*c*-Fe_7_Se_8_ (78%) whose X-ray diffraction (XRD) is shown in Fig. 1. All observed diffraction peaks in Fig. 1 index to either β-FeSe or 3*c*-Fe_7_Se_8_. 3*c*-Fe_7_Se_8_ is easily identified in the PLD target, instead of 4*c*-Fe_7_Se_8_, by the 3*c*-(115) peak at 2θ = 35.41°. The (115) reflection is due to the iron vacancy ordering so it is not present in the fundamental NiAs structure (δ-FeSe) and there are no possible 4*c* reflections near the same location. During certain growth conditions, the resulting films took on a doubly epitaxial configuration in which both β-FeSe and Fe_7_Se_8_ grew epitaxially oriented. β-FeSe was *c*-axis oriented, with the (001) plane oriented parallel to the substrate surface. Rocking curve analysis (Fig. 2) of the (001) reflection indicates mosaic structure in this phase, with a FWHM = 1.30° that is much larger than the instrumental resolution of 0.08°.

It cannot be assumed that 3*c*-Fe_7_Se_8_ formed during PLD growth because the specific structure of Fe_7_Se_8_ is highly dependent on growth conditions. Because 3*c*- and 4*c*-Fe_7_Se_8_ share the same fundamental NiAs-type structure, their powder XRD patterns differ only in vacancy superstructure diffraction peaks. Standard θ-2θ scans do not provide enough information to differentiate between the two structures when they are epitaxially oriented because the orientation makes many reflections geometrically unavailable. Based on the θ-2θ XRD scans in Fig. 1 of [1], the orientation of the Fe_7_Se_8_ phase was found to take on two different orientations with (101) and (001) planes oriented parallel to the substrate surface, using Miller indices referred to the setting of the fundamental NiAs-type structure of Fe_7_Se_8_. This convention of indexing the Fe_7_Se_8_ lattice planes and reflections with respect to its fundamental NiAs-type structure is adopted throughout this paper, unless otherwise noted, and is necessary whenever it is not possible to specify which Fe vacancy superstructure (3*c* or 4*c*) is present, which is our case.

**Fig. 3:**
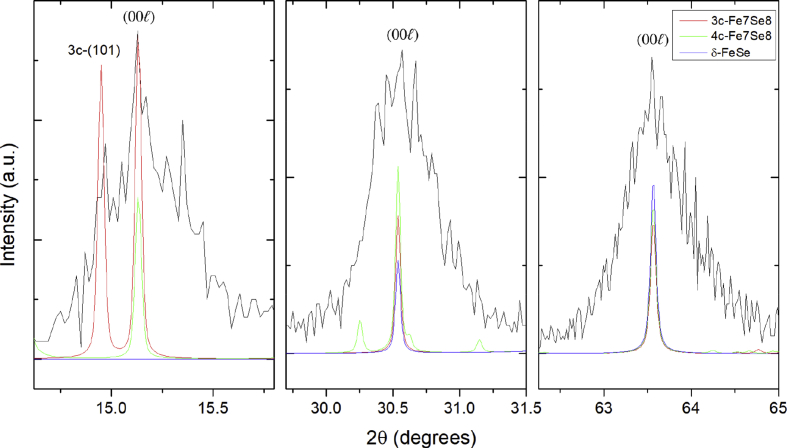
Detailed view of the *c*-axis Fe_7_Se_8_ peaks in the θ*-2*θ scan of the film grown at 550 °C and 3.4 J/cm^2^. Calculated XRD patterns of 3*c*, 4*c*, and δ-FeSe are overlaid to aid in determining the orientation of this phase.

In order to verify the (001) orientation of Fe_7_Se_8_, powder diffraction patterns were generated to compare with the θ-2θ scan of a thin film grown with a substrate temperature of 550 °C and laser fluence of 3.4 J/cm^2^, in which the *c*-axis diffraction peaks were more intense than in any other sample. Fig. 3 shows a detailed view of each of the Fe_7_Se_8_ (00l) peaks for this film, overlaid with the calculated diffraction patterns. At the lowest angle, the (001) Fe_7_Se_8_ reflection is observed at 2θ = 15.13° and is equivalent to the 3*c*-(003) and 4*c*-(004) reflections in the settings of the 3*c* and 4*c* structures, respectively. The observation of this peak rules out δ-FeSe for the *c*-axis orientation because the (001) reflection does not exist without the presence of ordered Fe vacancies. The next two peaks at 2θ = 30.54° and 2θ = 63.56° confirm the *c*-axis orientation, matching to the (002) and (004) Fe_7_Se_8_ reflections. The equivalent peak indices in the setting of their own crystal structures are (006) and (00.12) for 3*c*-Fe_7_Se_8_, and (008) and (00.16) for 4*c*-Fe_7_Se_8_. It should be noted that further information is required to differentiate between 3*c* and 4*c*.

2θ scans with ω = 2° were employed to search for additional diffraction peaks that could be used to verify the Fe_7_Se_8_ (101) orientation for films grown with substrate temperatures in the 350–450 °C range at a fixed laser fluence of 3.4 J/cm^2^. The relative fraction of β-FeSe to Fe_7_Se_8_ in these films changed from majority β-FeSe at 350 °C to majority Fe_7_Se_8_ at 450 °C. In 2θ scans, observed diffraction peaks correspond to crystal planes that are tilted with respect to the surface normal with an angle given by φ=θ−ω, where θ is the Bragg angle and ω is the incident angle of the x-rays. The angle between the crystal orientation and other diffraction planes, the interplanar angle, can be calculated to determine what angle ω is required to detect other diffraction planes in 2θ scans.

**Fig. 4:**
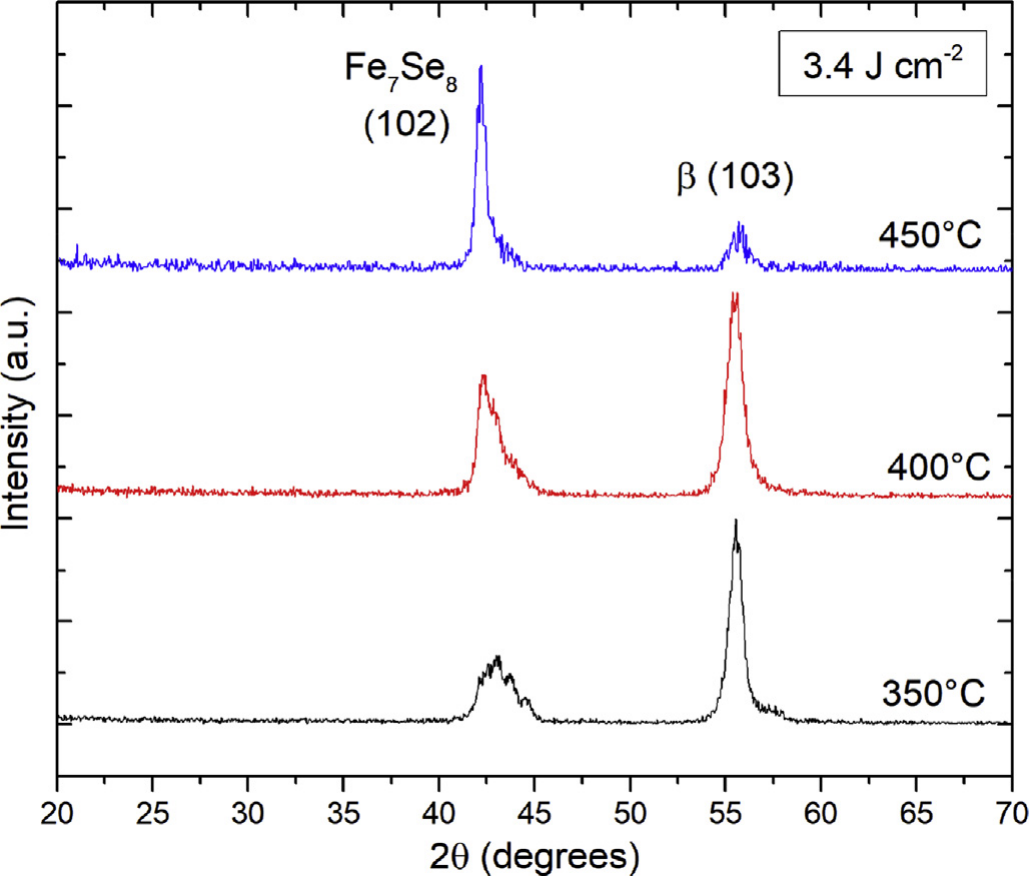
XRD 2θ scans (ω = 2°) of iron selenide films grown at different temperatures and laser fluence of 3.4 J/cm^2^. Scans confirm the Fe_7_Se_8_ (101) and β-FeSe (001) orientations.

In Fig. 4, the 2θ scans predominantly feature two major reflections, one near 2θ = 42.5° and the other near 2θ = 55.5°. The peak near 2θ = 42.5° is the (102) reflection of Fe_7_Se_8_, which is equivalent to either 3*c*-(206) or 4*c*-(408). The angle of this measured plane with respect to the substrate surface is 19.2° which is a good match to the 18.8° interplanar angle between Fe_7_Se_8_ (101) and (102), confirming the (101) orientation of Fe_7_Se_8_. The second major peak near 2θ = 55.5° is consistent with the β-FeSe (103) reflection, having an interplanar angle between β-FeSe (001) and (103) of 26.6°, which is a close match to the observed 25.8° with respect to the substrate surface. Additionally, the Fe_7_Se_8_ (103) reflection is expected at 55.5° and will be convoluted with β-(103). The interplanar angle for Fe_7_Se_8_ (103) with respect to (101) is 29.9° which is several degrees beyond what the 2θ scan should detect. This means that the majority, if not all, of the intensity measured near 2θ = 55.5° is due to the β-FeSe (103) reflection. Discrepancies between interplanar angles and 2θ positions are due to differences in the theoretical lattice constants used for calculations and the lattice constants of the actual thin film. The choice of ω = 2° is a compromise that enables both Fe_7_Se_8_ (102) and β-FeSe (103) to be visualized on the same XRD scan. Since mosaicity is confirmed in the films, the peaks observed in the 2θ scans are actually observable over a range of ω with the true peak intensity existing at some optimized ω value for each phase, which is unlikely to be exactly 2°. Therefore, the presented 2θ scans should not be used to calculate lattice constants because the peak 2θ value may be false. Reciprocal space mapping would enable the identification of the true peak intensity and correct lattice constants could be calculated.

The raw data for all of the XRD scans that were discussed have been uploaded alongside the article to be made available for download.

## 2 Experimental design, materials, and methods

Rocking curve and 2θ XRD scans were carried out on a Philips X'Pert-MPD with Cu Kα radiation. Incident and diffracted optics, as well as scan parameters were the same in each case. A 1/8° divergence slit and 10 mm mask were used on the incident side and the diffracted x-rays were passed through a parallel plate collimator and detected with a proportional counter. The step size was 0.05° with a time per step of 0.5 s. The incident angle for the 2θ scans was fixed at ω = 2° and 2θ was fixed at 16.064° for the rocking curve scan. PLD target composition was calculated using the Rietveld refinement function in Powder Cell [6].

The powder diffraction patterns for δ-FeSe, 3*c*-, and 4*c*-Fe_7_Se_8_ were generated using the VESTA software [7]. The 4*c* unit cell was defined in VESTA based on the crystal structure given by Okazaki [5] and the 3*c* structure was adapted from Parise [8] to have the lattice parameters *a* = 7.2631 Å and *c* = 17.550 Å. The 4*c* structure was made orthorhombic with lattice parameters *a* = 12.580 Å, *b* = 7.263 Å, and *c* = 23.400 Å. The 3*c* and 4*c* lattice parameters correspond to a fundamental NiAs-type structure with *a* = 3.632 Å and *c* = 5.850 Å. Lattice constants used for β-FeSe are *a* = 3.672 Å and *c* = 5.513 Å. Interplanar angles were calculated for Fe_7_Se_8_ with equation (1) and for β-FeSe with equation (2) [9].

(1) $$cos\varphi =\;\frac{h_{1}h_{2}+k_{1}k_{2}+\frac{1}{2}\left(h_{1}k_{2}+h_{2}k_{1}\right)+\frac{3}{4}\frac{a^{2}}{c^{2}}l_{1}l_{2}}{\sqrt{\left(h_{1}^{2}+k_{1}^{2}+h_{1}k_{1}+\frac{3}{4}\frac{a^{2}}{c^{2}}l_{1}^{2}\right)\left(h_{2}^{2}+k_{2}^{2}+h_{2}k_{2}+\frac{3}{4}\frac{a^{2}}{c^{2}}l_{2}^{2}\right)}}$$

(2) $$cos\varphi =\frac{\frac{h_{1}h_{2}+k_{1}k_{2}}{a^{2}}+\frac{l_{1}l_{2}}{c^{2}}}{\sqrt{\left(\frac{h_{1}^{2}+k_{1}^{2}}{a^{2}}+\frac{l_{1}^{2}}{c^{2}}\right)\left(\frac{h_{2}^{2}+k_{2}^{2}}{a^{2}}+\frac{l_{2}^{2}}{c^{2}}\right)}}$$
